# Simulation of the Metabolic Response to an Interventional Study with New Healthy Beverages by Machine-Learning Regression

**DOI:** 10.1021/acs.jafc.5c15421

**Published:** 2026-03-14

**Authors:** Diego Hernández-Prieto, Jose A. Egea, Cristina García-Viguera, Alberto Garre

**Affiliations:** † Lab Fitoquimica y Alimentos Saludables (LabFAS), CEBAS-CSIC, Campus Universitario Espinardo 25, 30100 Murcia, Spain; ‡ Group of Fruit Breeding, Department of Plant Breeding, CEBAS-CSIC, Campus Universitario de Espinardo 25, 30100 Murcia, Spain; § Associated Unit of R&D and Innovation CEBAS-CSIC+UPCT on “Quality and Risk Assessment of Foods”, Campus Universitario Espinardo 25, 30100 Murcia, Spain; ∥ Departamento de Ingeniería de Alimentos y del Equipamiento Agrícola, Instituto de Biotecnología Vegetal, Universidad Politécnica de Cartagena (ETSIA), Paseo Alfonso XIII, 48, 30203 Cartagena, Spain

**Keywords:** regression modeling, metabolic trial simulation, machine learning, natural beverages, personalized nutrition, nutritional analysis

## Abstract

The present study proposes a methodology to emulate an interventional trial by employing machine-learning (ML) models. A maqui-citrus beverage is used as a case study, exploiting empirical data to assess the performance of multiple ML algorithms, to further build regression models. Those models predicted the effect of consuming the beverage for 60 days, sweetened with different sweeteners, on flavanones and their metabolites and anthocyanin metabolites present in plasma and urine. To guarantee the reliability of the predictions, a comprehensive data analysis and preprocessing was carried out, followed by a hyperparameter tuning using Bayesian optimization. The models were benchmarked, yielding a goodness of fit *R* ^2^ of approximately 89% and reaching error rates (mean absolute error and root-mean-squared error) of about 2% and 10%, respectively. This study demonstrates the reliability of ML tools in simulating interventional trials, providing results without the need to expose participants to the intervention.

## Graphical abstract

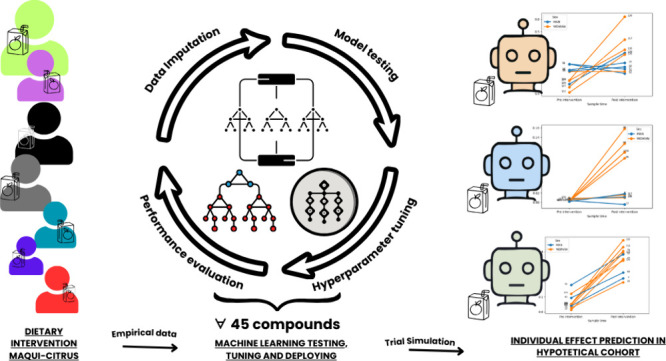

## 1 Introduction

Recent years have seen a notable increase in collaborative research endeavors between the fields of food and computer science. Advances in computational methods, such as machine learning (ML), have fostered our understanding of the biological processes associated with the metabolism of bioactive molecules present in functional foods and their effects on human health.^1,2^ There has been a gradual increase in the utilization of modeling and simulation in this area.^3–5^ Particularly, the development of metabolomic techniques that integrate ML models has enabled researchers to explore the complexities of food composition.^6–8^

Several studies have focused on examining and implementing data analytics in food sciences but mainly concerning structural dynamics,^9,10^ image recognition,^11,12^ metabolomics signal clarification,^13^ microbiological risk,^14^ and microbiota–food interactions.^15^ The employment of ML models has been instrumental in transforming research in numerous domains, including drug discovery,^16^ genomics, and personalized medicine,^17^ thus unveiling a vast array of possibilities. However, studies that apply predictive models to simulate interventional trials in food science are still scarce. Those models could be of great interest for the field because (longitudinal) interventional trials are still fundamental for nutritional studies.^18,19^ Predictive models could provide almost instantaneous estimates for the result of a potential intervention without requiring that participants are exposed to the product. This could foster research and development in the field, with predictive models providing preliminary results and trials rather being used as model validation.

The present study illustrates a methodology to develop ML models that assess the impact of a functional food on human physiology. This methodology was first outlined in part in the thesis of one of the authors.^20^ Namely, the models predict the concentrations of flavanones and their metabolites and anthocyanin metabolites present in blood and plasma following an intervention consisting of the consumption of a maqui-citrus beverage.^21^ The predictions are performed in relation to the baseline metabolite profile after a fixed period of time (60 days). The methodology includes the comparison of ML algorithms based on their ability to forecast the bioactive compound concentration after the intervention.

The models are developed using a data set from a previously published longitudinal trial^21^ that examined the influence of alternative sweeteners such as sucralose and stevia, heralded as healthier drink substitutes to soft drinks.^22,23^ That trial collected data on the concentrations of metabolites present in plasma and urine before and after the intervention. The study used a maqui-citrus beverage, designed to be rich in flavanones and other phenolic compounds with potential health benefits, including anthocyanins, which have attracted significant attention for their antioxidant, anti-inflammatory, and other bioactive properties.^24,25^ Furthermore, the study accounted for sex differences, an aspect whose importance is gaining much attention.^26,27^

In summary, the present study focuses on the implementation of ML models for forecasting how the consumption of a maqui-citrus beverage impacts the metabolite content in urine and plasma of participants, accounting for the effect of sweetener and sex. This approach is conducive to the investigation of healthier alternatives to high-sucrose soft drinks and the harnessing of the potential advantages of polyphenols in the beverages. Utilizing data from an interventional trial and employing sophisticated modeling techniques, the objective is to aid the advancement of reliable and effective forecasting tools that can contribute toward the development of personalized nutrition.

## 2 Materials and Methods

### 2.1 Empirical Data

ML models were developed by using the raw data from a previously published interventional trial. The trial was conducted on 138 overweight human individuals who consumed fresh maqui-citrus beverages on a daily basis for a duration of 60 days. Urine and plasma samples were collected at the commencement (day 0) and conclusion (day 60) of the intake period. The identification and quantification of phenolic compounds present in the urine and plasma samples were performed using high-performance liquid chromatography coupled with electrospray ionization triple quadrupole mass spectrometry.^21^ The resulting compounds were categorized into two groups: flavanones and their metabolites and anthocyanin metabolites. The concentration of each compound was inferred using calibration curves, and the results are expressed in ng/mL. A comprehensive list of the compounds identified can be found in Supplementary Table 1.

### 2.2 Data Imputation by Iterative Imputation

The raw data from the interventional study exhibited multiple missing values (due to participants opting out during the study or errors in quantification). This is a matter that arises in the training ML algorithms, which frequently proves to be incompatible with missing values. As a consequence, complete observations in which a single value is missing must be excluded. The task of filling in the missing values is known as imputation, and it was approached through utilization of the *IterativeImputer* tool implemented within the scikit-learn Python package, which models the missing value from a feature as a function of the other features considered, using a predictive regression algorithm. The technique iterates over each feature to be imputed until a prefixed number of iterations is completed or an early stopping criterion is met. This approach draws inspiration from the MICE R package,^28,29^ yet it employs a singular methodology, as opposed to a multiple one. The estimator employed was the implementation of the gradient boosting algorithm XGBoost (eXtreme Gradient Boosting algorithm).^30^ The process of imputation was conducted for each considered target variable, with its corresponding predictors defined in Section 2.3.1. The maximum number of iterations was set at 15, considering an early stopping when the relative change in the current iteration fell below the 10^–6^ threshold. The relative change was defined as the ratio between the maximum absolute change between the current iteration and the previous iteration and the maximum absolute value among the known values, utilized in this context as an escalating factor.

### 2.3 Model Building for the Impact of a Dietary Intervention on Participants

#### 2.3.1 Overall Approach

The objective of this study is to forecast the effect of the interventional trial on the composition of urine and plasma of the participants. Therefore, ML models were defined as a regression model, with the output variable being the concentration at the end of the intervention of each bioactive compound. The predictor variables were the vector of metabolite concentration prior to the intervention, plus the participant’s sex and the sweetener used in the beverage, as is summarized in Figure 1.

**Figure 1:**
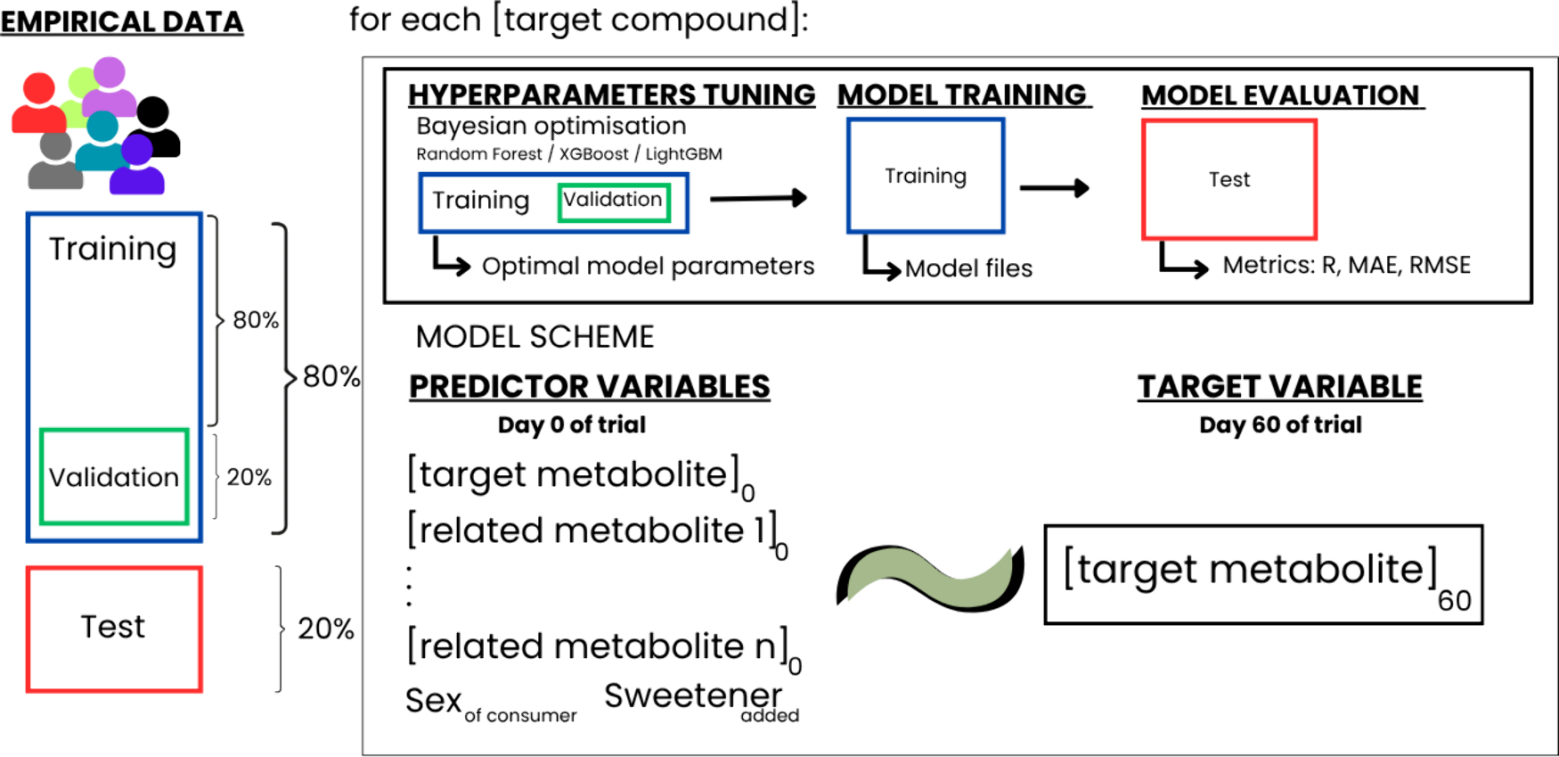
ML regression scheme for the simulation carried out.

The bioactive compounds of interest were selected due to their biomarker characteristics and potential health benefits (Table 1). They included VA, DHPAA, N, E, and HE, as well as their derivatives. The models also consider CA, TFA-S, and TFA-G, which were determined to show different bioavailability depending on the sex of the consumer.^31,32^ In addition, those studies found that E-S, HE-G, N-G, and DHPAA, which were already considered in the data set, were also differentially bioavailable by sex.

**Table 1:** Families of Bioactive Compounds Selected as Targets in the Regression Scheme and Their Properties

family of compounds	compounds	properties	sources
DHPAA	DHPAA, DHPAA-G, DHPAA-GG, DHPAA-GS, DHPAA-SS, Total DHPAA	anti-inflammatory, antioxidant, antibacterial, anticancer, and antiaging effects	^33^
VA	VA, VA-S, VA-GG, VA-SS, VA-GS, Total VA	anti(neuro)inflammatory and cardiovascular improvement effects	^34^
N	N, N-G, N-GG, N–S, Total N	anti-inflammatory and antioxidant effects	^35^
E	E, E-G, E-S, Total E	anti-inflammatory, antioxidant, anticancer, neuroprotective, cardioprotective, antidiabetic, and antiobesity effects	^36^
HE	HE, HE-G, HE-GG, Total HE	antioxidant and antinociceptive effects	^37^
additional metabolites	CA, TFA-S, TFA-G	selected due to previous results showing that their bioavailability varies between sexes	^31^,^32^

#### 2.3.2 Biological Constraints Introduced in the Modeling

On a first approximation, every metabolite was used as a predictor for the ML models. However, the resulting models tended to overfit, exhibited low performance, and demanded excessively long training times, due to the high model complexity (not shown). Therefore, the model complexity was reduced by employing as predictors only compounds that were biochemically related to the target compound being estimated. The process was initiated by considering the biochemical families for both samples (if available), and if the result was unsatisfactory, only the compounds from the same sample were employed as predictors (Table 2). The alignment of features with biological plausibility in the models resulted in enhanced predictive accuracy and interpretability. Furthermore, when redundant or irrelevant predictors were eliminated, the model complexity was reduced, lowering the risk of overfitting.

**Table 2:** Relationship of Predicted Metabolites to Their Predictor Features in the Models Presented

target metabolite	predictors
VA (urine), VA (plasma), VA-SS (urine), VA-SS (plasma), VA-GS (plasma), Total VA (urine), Total VA (plasma)	VA (urine), VA-GG (urine), VA-GS (urine), VA-SS (urine), Total VA (urine), VA (plasma), VA-GG (plasma), VA-S, VA-GS (plasma), VA-SS (plasma), Total VA (plasma)
TFA-S (urine), TFA-G (urine), Total TFA (urine), Total TFA (plasma)	TFA-G (plasma), TFA-S (plasma), Total TFA (plasma), TFA-G (urine), TFA-S (urine), Total TFA (urine)
HE, HE-G (urine), HE-G (plasma), HE-GG, Total HE	HE-G (plasma), HE, HE-G (urine), HE-GG, Total HE
N, N-G (urine), N-G (plasma), N–S, Total N	N-G (plasma), N, N-G (urine), N-GG, N-S, Total N
E (urine), E (plasma), E-G	E (plasma), E-S (plasma), E (urine), E-S (urine), E-G
CA (urine), CA (plasma)	CA (plasma), CA-G (plasma), CA-S (plasma), Total CA (plasma), CA (urine), CA-G (urine), CA-S (urine), CA-GS, Total CA (urine)
VA-GS (urine), VA-GG (urine)	VA (urine), VA-GG (urine), VA-GS (urine), VA-SS (urine), Total VA (urine)
DHPAA (urine), DHPAA (plasma), DHPAA-SS (urine), DHPAA-G (urine), DHPAA-G (plasma), DHPAA-GS (urine), DHPAA-GS (plasma), DHPAA-GG (plasma), Total DHPAA (urine), Total DHPAA (plasma)	DHPAA (plasma), DHPAA-G (plasma), DHPAA-GG (plasma), DHPAA-GS (plasma), DHPAA-SS (plasma), Total DHPAA (plasma), DHPAA (urine), DHPAA-G (urine), DHPAA-GG (urine), DHPAA-GS (urine), DHPAA-SS (urine), Total DHPAA (urine)
VA-S, VA-GG (plasma)	VA (plasma), VA-GG (plasma), VA-S, VA-GS (plasma), VA-SS (plasma), Total VA (plasma)
E-S (plasma)	E-S (plasma), E (plasma), Total E (plasma)
E-S (urine)	E (urine), E-S (urine)
Total E (urine)	E-G, Total E (urine)
TFA-S (plasma), TFA-G (plasma)	TFA-G (plasma), TFA-S (plasma), Total TFA (plasma)
N-GG	N, N-G (urine), N-GG, N-S, Total N

#### 2.3.3 ML Algorithms Considered

In preliminary calculations, different prediction methods such as Lasso, Ridge, and Elastic net regression and different ML algorithms were tested. After these preliminary tests, three state-of-the-art ML algorithms were compared in the study:^20^

(i) **Random Forest (RF):** This method, which belongs to the ensemble learning family, combines multiple, uncorrelated decision trees with the objective of improving the model’s overall performance.^38^ Each decision tree in the forest is trained on a different subset of the training data, and the final prediction of the model is made by aggregating the predictions of the individual trees. This approach helps to reduce overfitting and improve the generalization ability of the model.

(ii) **eXtreme Gradient Boosting (XGB):** Conceived as an implementation of the gradient-boosting ML algorithm,^30^ this method combines multiple weak, lower-accuracy models to enhance the overall performance of the model. Each weak model is trained sequentially to estimate the residual errors of the preceding weak model.

(iii) **Light Gradient-Boosting Machine (LGBM):** This is another implementation of the gradient-boosting algorithm, with higher efficiency and scalability in design.^39^ Based on a leaf-wise strategy, the LGBM builds decision trees, growing the tree vertically and prioritizing the leaves with the highest loss reduction and reducing training time while maintaining high predictive power.

#### 2.3.4 Model Training and Hyperparameter Tuning

The raw data were divided randomly, leaving 20% of the observations as the test set. These observations were excluded from training and hyperparameter tuning. This methodological decision was made with the objective of ensuring that the evaluation conducted with this set would be unbiased by the preceding phases. The remaining data were further divided into validation (20%) and training (80%) sets. The corresponding distribution of sex in the sets was 46 men and 36 women for the training set, 9 men and 13 women for the validation set, and 19 men and 9 women for the test set. Lastly, to enhance the performance and facilitate further comparisons, data were scaled in the range 0.1, employing the MinMaxScaler from scikit-learn.

Hyperparameters control the overall behavior of the model, such as the number of trees in RF or the tree depth.^40^ Hyperparameter tuning was done by Bayesian optimization using the Python framework *Optuna*.^41^ The Bayesian optimization is a method to determine optimal values for hyperparameters by estimating their performances within a probabilistic model, iterating to refine this model supported by observed results. Unlike other methods, such as grid search, which methodically explores all potential combinations, Bayesian optimization intelligently investigates the hyperparameter space, balancing exploration and exploitation to identify the optimal set of hyperparameters with a reduced number of evaluations.^20,42^ This approach has been shown to significantly reduce the computational cost and time required to optimize the model. *Optuna* requires the definition of feasible ranges for the hyperparameters, which were determined by preliminary simulations. The list of hyperparameters considered for each algorithm and their range is provided in Supplementary Table 2. The three ML algorithms (RF, XGB, and LGBM) were tuned and trained independently for each compound of interest (Table 2). This resulted in a total of 135 models.

#### 2.3.5 Evaluation of the Model Performance

The evaluation of the model’s performance was conducted by employing the mean absolute error (MAE) metric because of its simplicity and robustness to outliers and scale-dependency,^43^ and the root-mean-squared error (RMSE). MAE is defined as the average of the residuals (i.e., absolute differences between the actual values *y*_*i*_ and the predicted values *ŷ*_*i*_) provided by the ML algorithm, as shown in eq 1, where *n* is the number of observations. RMSE represents the standard deviation of the residuals of the predictions (eq 2). The units of MAE and RMSE in this context are nanograms per milliliter (ng/mL), which represent the error in predicting the concentration of the compounds in the fluid sample. Additionally, the coefficient of determination (*R* ^2^) was used to assess the proportion of variance in the dependent variable explained by the model^44^ (eq 3).

1 $$\mathrm{MAE}=\frac{1}{n}\Sigma_{i=1}^{n}\left|y_{i}-{ŷ}_{i}\right|$$

2 $$\mathrm{RMSE}=\sqrt{\frac{1}{n}\Sigma_{i=1}^{n}{\left(y_{i}-{ŷ}_{i}\right)}^{2}}$$

3 $$R^{2}=1-\frac{\Sigma_{i=1}^{n}{\left(y_{i}-{ŷ}_{i}\right)}^{2}}{\Sigma_{i=1}^{n}{\left(y_{\mathrm{mean}}-y_{i}\right)}^{2}}$$

#### 2.3.6 Simulation of a Hypothetical Cohort

A theoretical cohort simulation was conducted in order to illustrate how the developed models would assist in the design of dietary interventions. It was created by sampling a multivariate normal distribution based on the covariance and the mean of subsets corresponding to each combination of sex of the consumer and sweetener added to the beverage. From the six distributions, the trained models were used to predict the effect of the consumption of the maqui-citrus beverage over different consumers (Supplementary Figure 1). In order to provide a general overview of the impact of the intervention, the prediction variables were grouped according to bioactive compound families (Total VA and Total DHPAA in plasma and Total HE and Total N in urine). The variables Total VA and Total DHPAA were selected for quantification from plasma samples, while Total E and Total N were only quantified from urine samples.^20^ Although the results obtained for these scenarios should be taken with care (e.g., it does not account for sex differences in the initial metabolite concentration), it illustrates how the models could be used in an actual scenario.

### 2.4 Computational Methods

The computational procedures were carried out using Python version 3.11.7. The ML algorithms’ implementations used were the *xgboost* v.2.0.3 package^30^ for RF and XGB and the *lightgbm* v.4.3.0 package^39^ for LGBM. The process of Bayesian optimization for the purpose of tuning the model hyperparameters was conducted utilizing the *Optuna* v.3.6.1 package.^41^

## 3 Results and Discussion

### 3.1 Assessment of the Optimal Algorithm to Predict Each Metabolite Concentration

ML models were built using each algorithm proposed. Figure 2 illustrates the distribution of the MAE, RMSE, and *R* ^2^ of each method. Note that, because the models predict a family of compounds, the figures show the distribution over all of the compounds (Table 2). In general, XGB is the method with the most consistent results, displaying generally higher *R* ^2^ values (Figure 2A,D), with a median of 0.895 for plasma and 0.906 for urine, a lower quantile of 0.697 for plasma and 0.777 for urine, and an upper quantile of 0.961 for plasma and 0.959 for urine. In contrast, the values for *R* ^2^ for RF and LGBM were considerably lower, with a lower quantile of 0.461 and 0.602 for plasma and 0.460/0.602 for urine and an upper quantile of 0.887 and 0.868, respectively, for plasma and 0.887/0.868 for urine. A comparison between LGBM and RF shows that the former is generally more stable, with a lower dispersion.

**Figure 2:**
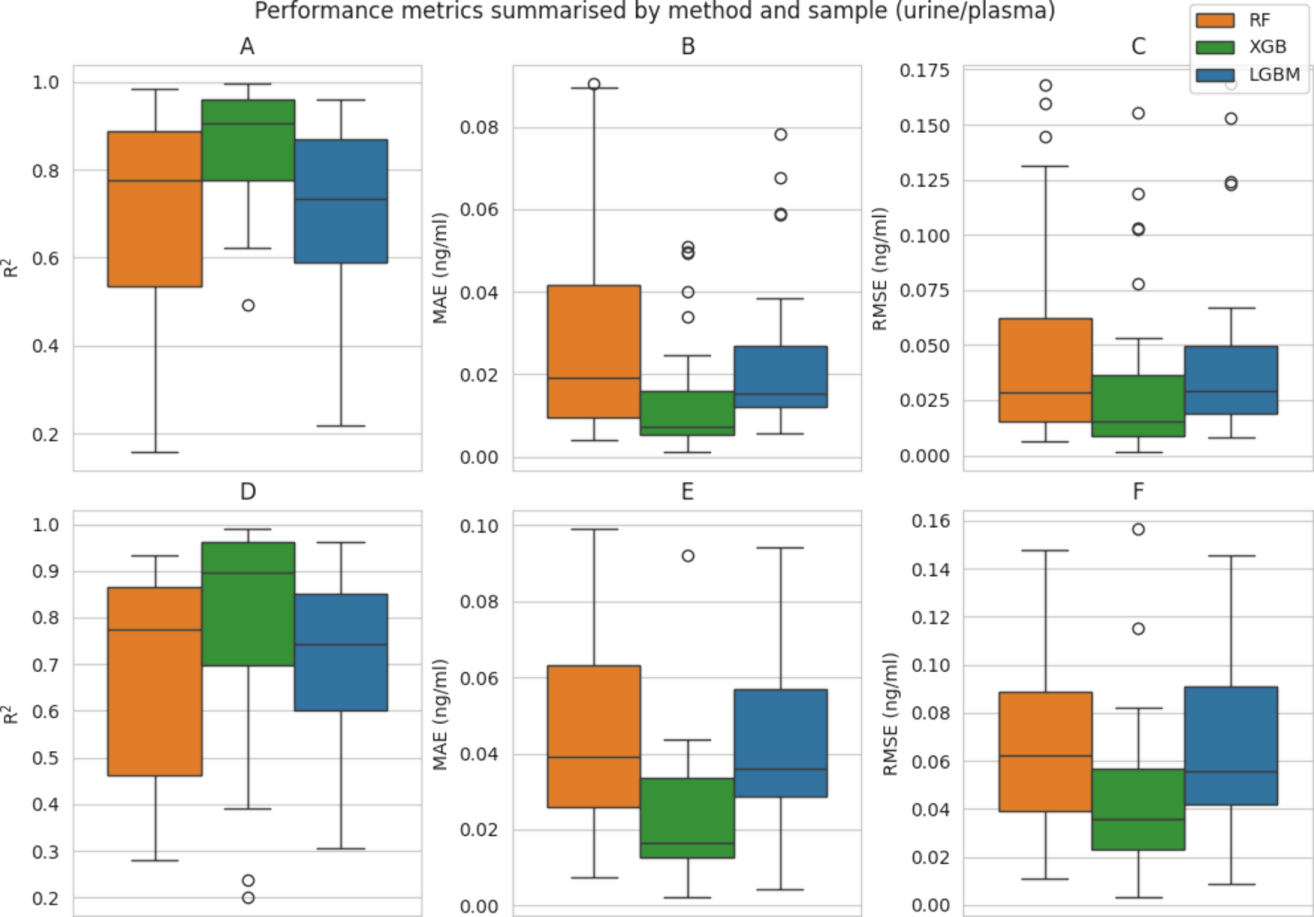
Boxplot of values from performance tests for evaluating the algorithms tested for each bioactive compound prediction. Summary of the results of (A and D) *R* ^2^, (B and E) MAE, and (C and F) RMSE.

As expected, MAE and RMSE (Figure 2B,C,E,F) followed a similar distribution as *R* ^2^. XGB had the greatest predictive power, with a median MAE of 0.0124 ng/mL, a lower quartile of 0.00564 ng/mL, and an upper quartile of 0.0256 ng/mL for plasma and a median of 0.0737 ng/mL, a lower quartile of 0.00524 ng/mL, and an upper quartile of 0.0160 ng/mL for urine. RMSE values obtained by XGB showed a median of 0.035 ng/mL (plasma) and 0.015 ng/mL (urine), a lower quartile of 0.00890 ng/mL (plasma) and 0.0231 ng/mL (urine), and an upper quartile of 0.0565 ng/mL (plasma) and 0.0362 ng/mL (urine). The MAE and RMSE values for RF and LGBM were once again more dispersed with higher medians (0.0284 and 0.0291 ng/mL, respectively, for urine, and 0.0623 and 0.0554 ng/mL, respectively, for plasma) and wider ranges for the lower and upper quartiles for each sample.

Although XGB showed an overall better predictive power than the other two ML algorithms (Supplementary Table 3), this case study requires forecasting the concentration of 47 individual compounds (Table 2). Therefore, instead of using a unique algorithm, the algorithm that best predicted each compound was selected based on MAE and *R* ^2^. This is most reasonable in our case because the suitability of each algorithm depends on the underlying (nonlinear) relationships of each system. It is likely that these are different for each compound (especially considering that each compound uses different inputs), so imposing the same ML algorithm for every compound seems unreasonable.

The distribution of which bioactive compounds were best predicted by XGB was not characterized by any distinguishable pattern. For the VA and its derivatives subset, the predictions made by XGB were consistently superior to those of the remaining algorithms. The CA family exhibited a mere two metabolites, thus not constituting a whole subgroup in the present analysis. The remaining subgroups exhibited at least two elements that were not optimally predicted by XGB. In view of the absence of any discernible pattern, further analyses are required to determine the most effective strategy for predicting different subgroups. This, in turn, should enhance the performance of a holistic prediction for the entire metabolic system under study.^20^

The application of best-fitted ML algorithms for individual bioactive compound prediction resulted in an *R* ^2^ higher than 0.7 for every compound, achieving values above 0.9 in 62% of the compounds. However, DHPAA-SS in plasma and DHPAA-GG in urine yielded lower values (0.59 and 0.66, respectively), thus rendering them “nonpredictable” by the ML models developed here. This could be due to the nature of these compounds, whose digestion is linked to microbial fermentation. Therefore, it would be ruled by factors (such as the gut microbiota) that are not considered by the model, and these two compounds were excluded from further analysis. A more profound examination of the associations between the two groups of compounds and the provenance of the samples revealed that both groups were most accurately predicted in urine samples, with slightly superior outcomes observed for flavanones and their metabolites (Supplementary Table 4 and Supplementary Figure 2).

### 3.2 Predicting the Outcome of Dietary Intervention Use on the Predictive Model

The predictive models developed here could be of great use in personalized nutrition because they can predict instantly the result of different interventions on a participant. As an illustration of this use, a hypothetical cohort was simulated. The predicted effect of consuming the maqui-citrus beverage on a daily basis over a period of 2 months is illustrated in Figure 3. In all of the simulated cases, the consumption of the beverage resulted in an overall increase in the bioavailability of bioactive compounds, with the exception of the men who consumed the beverage with sucralose, where the postintervention bioavailability predicted was on average lower than the preintervention. Moreover, the results demonstrate that there are differences by sex: women generally exhibited higher concentrations of Total N and Total HE in the urine. The findings are consistent with the conclusions previously obtained on research carried out with the same data set, drawn using classical statistical methods.^31,32^ This emphasizes that on a population level the predictions of ML models should generally align with the results of classical statistical analyses.

**Figure 3:**
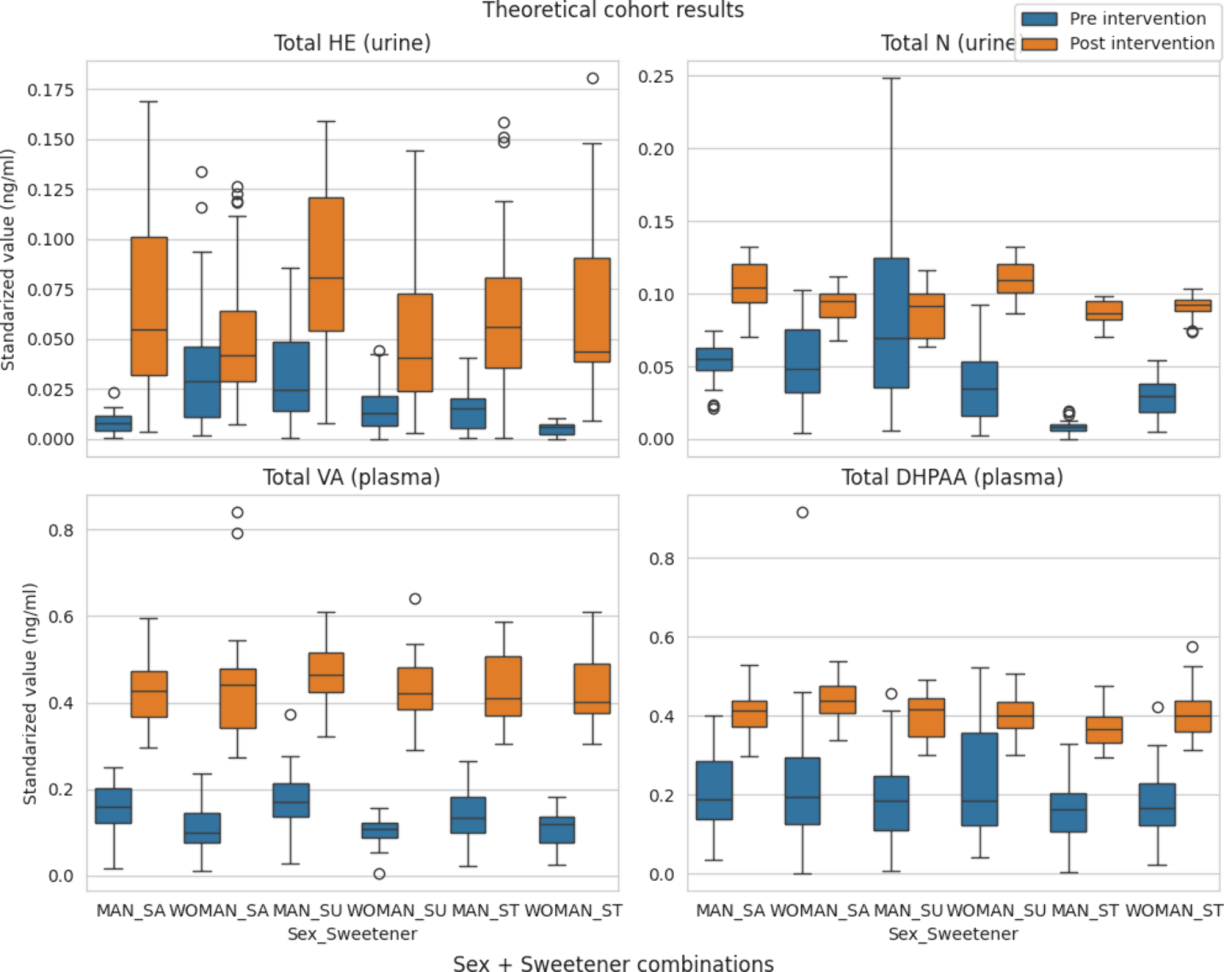
Boxplot showing the distribution of values of the variables aggregating metabolites: (blue) values of the new simulated consumers of the beverage; (orange) values predicted by the models.

The added value of ML models is their ability to predict an individualized response based on each participant’s attributes, something that is not possible using classical methods, which are often limited to general trends. As a demonstrative example, Figure 4 illustrates the predicted response for participants with the most extreme response, depicting the highest increases in the bioavailability. For instance, when observing the predictions for Total N (Figure 4A) of volunteers 19, 60, 69, 66, and 9, low to negative increases can be described. It is noteworthy that these subjects are all male with the exception of subject 66. Consequently, it can be deduced that, in the event of an objective being set to increase the levels of N and its relative compounds by a nutrition physician, the consumption of the maqui-citrus beverage should be avoided. The division in terms of sexes was very relevant in Total HE value predictions (Figure 4B), with the top five highest increases being women, while the top five lowest increases were four men and one woman. The differences were less evident in the Total DHPAA changes (Figure 4C), while in Total VA, the differences were evident, reaching cases like volunteers 11, 51 and 66, whose Total VA bioavailability apparently decreased.

**Figure 4:**
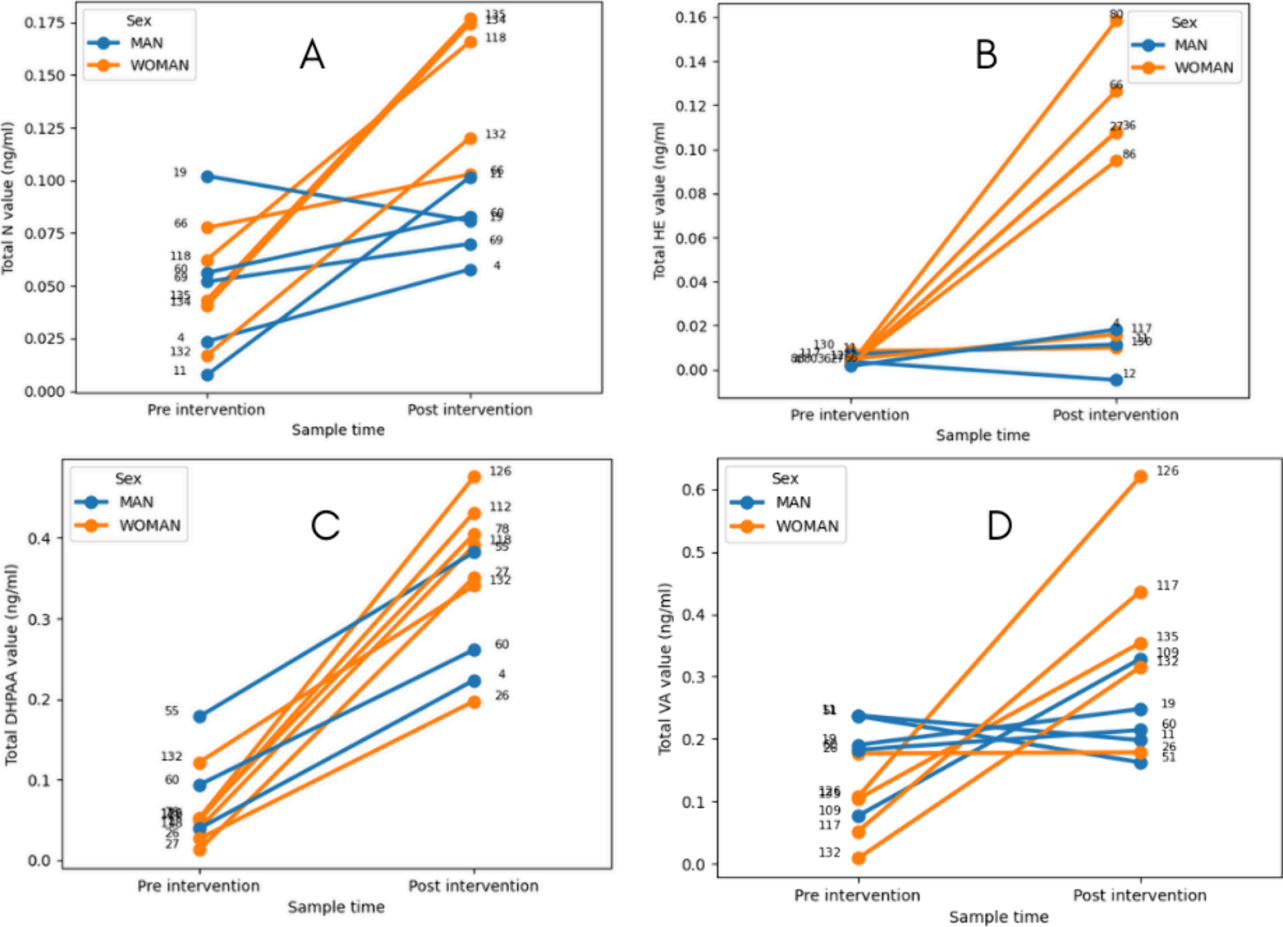
Point plot for the predicted increments in the bioavailability of bioactive compounds after beverage consumption by the virtual cohort. The predicted variables are (A) Total N in urine, (B) Total HE in urine, (C) Total DHPAA in plasma, and (D) Total VA in plasma.

These simulations illustrate the potential of these ML models as a valuable asset in practical scenarios. The development of a model of this nature for a range of interventions (e.g., diverse beverage recipes) is a conceivable prospect. Subsequently, considering the starting conditions of each potential consumer, it is possible to identify the intervention that is more suitable for each individual consumer. This would represent a substantial advancement in this domain, where the quantitative outcome of dietary interventions can only be ascertained *posthoc*. Nevertheless, further expansions of the presented computational tool remain in future experimental essays.

The principal aim of the present study was to evaluate the reliability of a number of ML algorithms for simulating the metabolic response to an interventional trial with maqui-citrus beverages. This involved predicting the effects of the consumption of the beverage on the bioavailability of several flavonoids and/or their metabolites, with consideration also given to the sex of the consumer and the sweetener added. Subsequently, individual models for each concentration of bioactive compound prediction were constructed to enable the full simulation to be completed. The hypothesis was that the selected algorithms, after being carefully tuned and including the appropriate target and predictor variables for each compound, would be capable of accurately simulating the results of an interventional trial with a new subject of study, following training of the models with data from empirical studies. The development of a precise and reliable computational method to simulate these trials represents a more cost- and time-efficient procedure for investigating the timewise effects of the consumption of novel food products. Furthermore, it enables future insights into personalized nutrition^45^ and novel approaches toward the understanding of the mechanism of human metabolism to incorporate (poly)­phenols to organism, measured in the bioavailability.

A variety of methodologies exist for the simulation of complex biological systems, characterized by numerous input and output variables.^46^ In the initial phases of the simulation’s development, multioutput regression was investigated as a potential optimal strategy. This paradigm encompasses a multitude of predictor variables, with each predictor associated with more than one target variable, designed to capture the correlation between the variables, whether they are output or input. It can be posited that, in principle, correlation information would facilitate a more precise prediction. Nevertheless, this methodology would necessitate a more computationally costly optimization and extended training times, and it has the potential to yield unrealistic results by establishing mathematically consistent relationships between variables whose associations are biologically implausible or extraneous. Ultimately, the selected approach, as an alternative to multioutput regression, was the deployment of a pipeline consistent with numerous independent single-output models, whose individual predictions were dependent on the compounds related to the target bioactive to be estimated after the intervention. The combination of all of the models’ outcomes enables the formulation of a general prediction of the metabolic response to the dietary intervention. The proposed method has been demonstrated to be effective in terms of prediction quality and accuracy.^20^ However, this approach does result in certain compromises in the fidelity of the simulated system because it does not accurately consider the potential interactions between target variables. Additional research may yield the optimal mechanism for predicting this scenario.

A salient feature of computational models grounded in ML pertains to the training, validation, and testing of said models, which is primarily informed by analogous experimental data. Consequently, the accuracy of the predictions made by an ML algorithm is contingent upon the quality of the experimental data upon which it is based. Upon initial consideration, this may appear to be a relatively insignificant limitation in the field of food science, where interventional nutritional trials are frequently carried out.^20^ This may suggest that a significant quantity of data sets, containing results from the aforementioned trials, would be accessible and appropriate for training, testing, and validation of the computational models.^47^ Conversely, attaining data that are both useful and accessible is challenging because the majority of such data sets are not available via Open Access and/or employ experimental procedures that are not analogous. Moreover, a bias in the experimental design has been observed, with certain demographics exhibiting underrepresentation.^48,49^ It is evident that this demonstrates a significant limitation when attempting to extrapolate the results to a broader, more heterogeneous population. Consequently, it is challenging to test the presented simulation in real-world scenarios.^50^ The applicability of the models developed in this work to different populations or dietary interventions is limited. Indeed, responses of (perhaps distinct) bioactive compounds relevant in other studies of this kind could exhibit different behavior and/or dynamics of those considered here. Nevertheless, for the purpose of designing dietary interventions employing the same types of beverages and populations, it is anticipated that the presented models will prove to be powerful tools.

In instances in which openly available databases are not accessible, it is incumbent upon each computational study to include a sufficient number of repeated trials for each food product. There are ways to overcome these challenges. One possible approach to address this issue is to integrate existing knowledge about the human metabolism response to the consumption of different bioactive compounds under different conditions. This would serve to reduce the reliance on particular experimental data, thereby improving the reliability and flexibility of the simulation. An alternative approach involves the reconstruction, facilitation, and curation of databases of metabolic responses to nutritional interventions.^51^ This would facilitate the design and execution of a greater number of models in numerous research groups because greater accessibility of data empowers researchers to conduct novel studies and provide new replications of already published ones.^20^ With regard to the present study, no other research has hitherto been found that has evaluated an intervention involving a maqui beverage on an independent cohort. Consequently, the independent test set is regarded as the most appropriate metric for evaluating the model’s predictive capability.

The primary constraint limitation derived from the empirical data was the absence of intermediate sampling points, thus precluding the utilization of forecasting models, which necessitate multiple sampling points in addition to initial and final measurements. Forecasting models would have facilitated predictions extending beyond the 60-day period, encompassing a more substantial time span for the simulation scenarios.^52^ Further research could include the weighted action of the anthropometric and health index values, but currently it is difficult to investigate because the interpretability of ML models in biology is a field in the early development stage.^53,54^

Finally, anthropometric values and cardiovascular health indexes were incorporated as potential predictor variables into the simulation during the initial stages of models’ development. However, the relative importance of these variables in the prediction result obscured the effect of the other predictor variables (specifically, the bioactive compounds’ concentrations). Consequently, they were removed in the pursuit of simpler, bioactive-focused models. This could have been caused by the fact that these measures have a signal-to-noise ratio much higher than that of the nutritional compounds. Although including these variables would certainly improve the model predictive capacity, the methodologies to do so are certainly complex, so this is left for future research.

In summary, the study’s primary conclusion assessed the capacity of ML models to simulate an interventional longitudinal trial involving a maqui-citrus-based beverage, incorporating factors such as the sweetener used and the consumer’s sex. For the bioactive compounds under consideration, MAE in the prediction represented an average error between 2% and 10%, the RSME exhibited errors between the 10% and 15%, while the coefficient *R* ^2^ exhibited a high value average, with at least 31 cases exceeding 0.9. XGB emerged as the best-performing algorithm, making it a reliable option for predicting all concentrations of compounds. However, LGBM remained a viable choice for faster and less computational-demanding executions.

Notwithstanding the challenges associated with simulating certain aspects of the biological system, the employment of meticulously tested and calibrated ML algorithms has been proven to facilitate a reliable simulation of the metabolic response to nutritional interventions. The findings obtained through this benchmarking process serve as a foundation for developing a user-friendly software tool that aims to assist noncomputational scientists in their predictions. In order to evaluate the limitations of these ML models, future research should test their performance with diverse and even implausible input data. It is imperative that this be done in order to guarantee that the models are capable of accommodating and moderating the results within a biologically reasonable margin of error. For future work, new modeling approaches should be formulated based on a more extensive range of foods, utilizing data sets derived from empirical studies. This approach will enable the generation of more generalizable models that could be employed to evaluate a growing array of healthy foods, thereby improving personalized nutrition.

## Supplementary Material



## Notes

Metabolites found and quantified in the interventional trial (Supplementary Table 1), list of hyperparameters considered in Bayesian optimization procedure to be tuned for each model with its range assessed (Supplementary Table 2), metrics obtained for the best performance model for every metabolite considered (Supplementary Table 3), evaluation of the effects of biochemical family and sample type factors (along with their interactions) on the accuracy obtained by studied models (Supplementary Table 4), descriptive statistical values for each concentration of bioactive compound (Supplementary Table 5), scheme of the virtual cohort for the simulation experiments (Supplementary Figure 1), and boxplot of values obtained in performance metrics by the two families considered (Supplementary Figure 2) (PDF)

## Abbreviations

CA: 3′,4′-dihydroxycinnamic acid
CA-G: CA-glucuronide
CA-GS: CA-glucuronide-sulfate
Total CA: total amount of CA and its derivatives
DHPAA: 3′,4′-dihydroxyphenylacetic acid
DHPAA-G: DHPAA-glucuronide
DHPAA-GS: DHPAA-glucuronide-sulfate
DHPAA-SS: DHPAA-disulfate
Total DHPAA: total amount of DHPAA and its derivatives
E: eriodyctiol
E-G: E-glucuronide
E-S: E-sulfate
Total E: total amount of E and its derivatives
HE: homoeriodyctiol
HE-G: HE-glucuronide
HE-GG: HE-diglucuronide
Total HE: total amount of HE and its derivatives
N: naringenin
N-G: N-glucuronide
N-GG: N-diglucuronide
N–S: N-sulfate
Total N: total amount of naringenin and its derivatives
TFA-G: 4′-hydroxy-3′-methoxycinnamic acid-glucuronide
TFA-S: 4′-hydroxy-3′-methoxycinnamic acid-sulfate
Total TFA: total amount of TFA and its derivatives
VA: 4-hydroxy-3-methoxybenzoic acid
VA-GG: VA-diglucuronide
VA-SS: VA-disulfate
VA-GS: VA-glucuronide-sulfate
Total VA: total amount of VA and its derivatives

## References

[1] Tseng Y. J., Chuang P.-J., Appell M. (2023). When Machine Learning and Deep Learning
Come to the Big Data in Food Chemistry. ACS
Omega.

[2] Zargar S., Wani T. A., Rizwan Ahamad S. (2023). An Insight into Wheat Germ Oil Nutrition,
Identification of Its Bioactive Constituents and Computer-Aided Multidimensional
Data Analysis of Its Potential Anti-Inflammatory Effect via Molecular
Connections. Life.

[3] Li S., Tian Y., Jiang P., Lin Y., Liu X., Yang H. (2021). Recent Advances in the Application of Metabolomics for Food Safety
Control and Food Quality Analyses. Critical
Reviews in Food Science and Nutrition.

[4] de
Souza T. A., Rodrigues G. C. S., de Souza P. H. N., Abreu L. S., Pereira L. C. O., da Silva M. S., Tavares J. F., Scotti L., Scotti M. T. (2023). Mass Spectrometry-Based Investigation of Sugarcane
Exposed to Five Different Pesticides. Life.

[5] Zhao H., Wang S. C. (2022). A Coding Basis and
Three-in-One Integrated Data Visualization
Method ‘Ana’ for the Rapid Analysis of Multidimensional
Omics Dataset. Life.

[6] Wang, Y. ; Yang, J. ; Yu, S. ; Fu, H. ; He, S. ; Yang, B. ; Nan, T. ; Yuan, Y. ; Huang, L. Prediction of Chemical Indicators for Quality of Zanthoxylum Spices from Multi-Regions Using Hyperspectral Imaging Combined with Chemometrics. Front. Sustainable Food Syst. 2022, 6. 10.3389/fsufs.2022.1036892.

[7] Fuentes S., Tongson E., Torrico D. D., Gonzalez
Viejo C. (2020). Modeling Pinot
Noir Aroma Profiles Based on Weather and Water Management Information
Using Machine Learning Algorithms: A Vertical Vintage Analysis Using
Artificial Intelligence. Foods.

[8] Gunaratne T. M., Gonzalez Viejo C., Gunaratne N. M., Torrico D. D., Dunshea F. R., Fuentes S. (2019). Chocolate
Quality Assessment Based on Chemical Fingerprinting
Using Near Infra-Red and Machine Learning Modeling. Foods.

[9] Tao X., Huang Y., Wang C., Chen F., Yang L., Ling L., Che Z., Chen X. (2020). Recent developments
in molecular docking technology applied in food science: a review. Int. J. Food Sci. Technol..

[10] Chen, F. ; Chen, Z. ; Sun, H. ; Zhu, J. ; Wu, K. ; Zhou, S. ; Huang, Y. Dendrobium Candidum Quality Detection in Both Food and Medicine Agricultural Product: Policy, Status, and Prospective. Front. Sustainable Food Syst. 2022, 6. 10.3389/fsufs.2022.1042901.

[11] Kang, H. ; Dai, D. ; Zheng, J. ; Liang, Z. ; Chen, S. ; Ding, L. Identification of Hickory Nuts with Different Oxidation Levels by Integrating Self-Supervised and Supervised Learning. Front. Sustainable Food Syst. 2023, 7. 10.3389/fsufs.2023.1144998.

[12] Setiadi, I. C. ; Hatta, A. M. ; Koentjoro, S. ; Stendafity, S. ; Azizah, N. N. ; Wijaya, W. Y. Adulteration Detection in Minced Beef Using Low-Cost Color Imaging System Coupled with Deep Neural Network. Front. Sustainable Food Syst. 2022, 6. 10.3389/fsufs.2022.1073969.

[13] Pomyen Y., Wanichthanarak K., Poungsombat P., Fahrmann J., Grapov D., Khoomrung S. (2020). Deep Metabolome: Applications of Deep Learning in Metabolomics. Computational and Structural Biotechnology Journal.

[14] Chen Q., Zhao Z., Wang X., Xiong K., Shi C. (2022). Microbiological
Predictive Modeling and Risk Analysis Based on the One-Step Kinetic
Integrated Wiener Process. Innovative Food Science
& Emerging Technologies.

[15] Sabater C., Calvete-Torre I., Villamiel M., Moreno F. J., Margolles A., Ruiz L. (2021). Vegetable Waste and
By-Products to Feed a Healthy Gut Microbiota:
Current Evidence, Machine Learning and Computational Tools to Design
Novel Microbiome-Targeted Foods. Trends in Food
Science & Technology.

[16] U.S. Government Accountability Office . Artificial Intelligence in Health Care: Benefits and Challenges of Machine Learning in Drug Development. Reissued with revisions on Jan 31, 2020. Available online at https://www.gao.gov/products/gao-20-215sp (accessed on 2023-11-23).

[17] Arora A., Olshen A. B., Seshan V. E., Shen R. (2020). Pan-Cancer Identification
of Clinically Relevant Genomic Subtypes Using Outcome-Weighted Integrative
Clustering. Genome Medicine.

[18] Thomas D. M., Kleinberg S., Brown A. W., Crow M., Bastian N. D., Reisweber N., Lasater R., Kendall T., Shafto P., Blaine R. (2022). Machine Learning Modeling
Practices to Support the
Principles of AI and Ethics in Nutrition Research. Nutr Diabetes.

[19] Ma C., Chen Q., Mitchell D. C., Na M., Tucker K. L., Gao X. (2022). Application
of the Deep Learning Algorithm in Nutrition Research
– Using Serum Pyridoxal 5′-Phosphate as an Example. Nutrition Journal.

[20] Hernández-Prieto, D. Computational Design of Beverages: Exploiting AI-Based to Obtain Healthier Drinks. Ph.D. Thesis, Universidad Politécnica de Cartagena, Cartagena, Spain, 2024. https://repositorio.upct.es/server/api/core/bitstreams/31f1d2ba-5231-4cb4-84dc-29c579c7462c/content.

[21] Agulló V., García-Viguera C., Domínguez-Perles R. (2021). Beverages
Based on Second Quality Citrus Fruits and Maqui Berry, a Source of
Bioactive (Poly)­Phenols: Sorting Out Urine Metabolites upon a Longitudinal
Study. Nutrients.

[22] Pepe R. B., Lottenberg A. M., Fujiwara C. T. H., Beyruti M., Cintra D. E., Machado R. M., Rodrigues A., Jensen N. S. O., Caldas A. P. S., Fernandes A. E. (2023). Position Statement on Nutrition Therapy
for Overweight and Obesity: Nutrition Department of the Brazilian
Association for the Study of Obesity and Metabolic Syndrome (ABESO-2022). Diabetol Metab Syndr.

[23] Escobar
Gil T., Laverde Gil J. (2023). Artificially Sweetened Beverages Beyond the Metabolic
Risks: A Systematic Review of the Literature. Cureus.

[24] Roszkowski S. (2023). Application
of Polyphenols and Flavonoids in Oncological Therapy. Molecules.

[25] Neyestani T. R., Yari Z., Rasekhi H., Nikooyeh B. (2023). How Effective Are Anthocyanins
on Healthy Modification of Cardiometabolic Risk Factors: A Systematic
Review and Meta-Analysis. Diabetol Metab Syndr.

[26] Holdcroft A. (2007). Gender bias
in research: how does it affect evidence based medicine?. Journal of the Royal Society of Medicine.

[27] Rodrigues H., Goméz-Corona C., Valentin D. (2020). Femininities & masculinities:
sex, gender, and stereotypes in food studies. Current Opinion in Food Science.

[28] Azur M. J., Stuart E. A., Frangakis C., Leaf P. J. (2011). Multiple imputation
by chained equations: what is it and how does it work?. International Journal of Methods in Psychiatric Research.

[29] Hernandez-Prieto D., García-Viguera C., Egea J. A. (2024). Enhancing statistics
and machine learning results from an interventional longitudinal dietary
study applying a data imputation system. Acta
Horticulturae.

[30] Chen, T. ; Guestrin, C. XGBoost: A Scalable Tree Boosting System. Proceedings of the 22nd ACM SIGKDD International Conference on Knowledge Discovery and Data Mining. KDD ’16: The 22nd ACM SIGKDD International Conference on Knowledge Discovery and Data Mining, San Francisco, CA; ACM, 2016; pp 785–794. 10.1145/2939672.2939785.

[31] Hernández-Prieto D., Fernández P. S., Agulló V., García-Viguera C., Egea J. A. (2023). Bioactive Compounds in Plasma as a Function of Sex
and Sweetener Resulting from a Maqui-Lemon Beverage Consumption Using
Statistical and Machine Learning Techniques. International Journal of Molecular Sciences.

[32] Hernández-Prieto D., Garre A., Agulló V., García-Viguera C., Egea J. A. (2023). Differences
Due to Sex and Sweetener on the Bioavailability
of (Poly)­phenols in Urine Samples: A Machine Learning Approach. Metabolites.

[33] Khan, A. K. ; Rashid, R. ; Fatima, N. ; Mahmood, S. ; Mir, S. ; Khan, S. ; Jabeen, N. ; Murtaza, G. Pharmacological activities of protocatechuic acid. Acta Polym. Pharm. 2015, 72 (4), 643–650. 26647619

[34] Sharma, N. ; Tiwari, N. ; Vyas, M. ; Khurana, N. ; Muthuraman, A. ; Utreja, P. An overview of therapeutic effects of vanillic acid. Plant Arch. 2020, 20 (2) 3053–3059.

[35] Rani N., Bharti S., Krishnamurthy B., Bhatia J., Sharma C., Kamal M. A., Ojha S., Arya D. S. (2016). Pharmacological
Properties and Therapeutic Potential of Naringenin: A Citrus Flavonoid
of Pharmaceutical Promise. Curr. Pharm. Des..

[36] Islam A., Islam M. S., Rahman M. K., Uddin M. U., Akanda M. R. (2020). The pharmacological
and biological roles of eriodictyol. Archives
of Pharmacal Research.

[37] Roohbakhsh A., Parhiz H., Soltani F., Rezaee R., Iranshahi M. (2014). Neuropharmacological
properties and pharmacokinetics of the citrus flavonoids hesperidin
and hesperetin — A mini-review. Life
Sciences.

[38] Rigatti S. J. (2017). Random
Forest. Journal of Insurance Medicine.

[39] Ke, G. ; Meng, Q. ; Finley, T. ; Wang, T. ; Chen, W. ; Ma, W. ; Ye, Q. ; Liu, T.-Y. LightGBM: A Highly Efficient Gradient Boosting Decision Tree. Proceedings of the Advances in Neural Information Processing Systems; Curran Associates, Inc, 2017; Vol. 30.

[40] Probst P., Boulesteix A.-L., Bischl B. (2018). Tunability: Importance of Hyperparameters
of Machine Learning Algorithms Ver. 3. arXiv.

[41] Akiba, T. ; Sano, S. ; Yanase, T. ; Ohta, T. ; Koyama, M. Optuna: A Next-Generation Hyperparameter Optimization Framework. Proceedings of the 25th ACM SIGKDD International Conference on Knowledge Discovery & Data Mining; KDD ’19; Association for Computing Machinery: New York, 2019; pp 2623–2631. 10.1145/3292500.3330701.

[42] Shahriari B., Swersky K., Wang Z., Adams R. P., de Freitas N. (2016). Taking the
Human Out of the Loop: A Review of Bayesian Optimization. Proceedings of the IEEE.

[43] Botchkarev A. (2019). Performance
Metrics (Error Measures) in Machine Learning Regression, Forecasting
and Prognostics: Properties and Typology. Interdisciplinary
Journal of Information, Knowledge, and Management.

[44] Chicco D., Warrens M. J., Jurman G. (2021). The Coefficient
of Determination
R-Squared Is More Informative than SMAPE, MAE, MAPE, MSE and RMSE
in Regression Analysis Evaluation. PeerJ. Comput.
Sci..

[45] Verma, M. ; Hontecillas, R. ; Tubau-Juni, N. ; Abedi, V. ; Bassaganya-Riera, J. Challenges in Personalized Nutrition and Health. Front. Nutrition 2018, 5. 10.3389/fnut.2018.00117. PMC6281760 30555829

[46] Peng G. C. Y., Alber M., Buganza Tepole A., Cannon W. R., De S., Dura-Bernal S., Garikipati K., Karniadakis G., Lytton W. W., Perdikaris P. (2021). Multiscale Modeling
Meets Machine Learning: What Can We Learn?. Archives of Computational Methods in Engineering.

[47] Rychlik M., Zappa G., Añorga L., Belc N., Castanheira I., Donard O. F. X., Kouřimská L., Ogrinc N., Ocké M. C., Presser K., Zoani C. (2018). Ensuring Food
Integrity
by Metrology and FAIR Data Principles. Frontiers
in Chemistry.

[48] Daitch V., Turjeman A., Poran I., Tau N., Ayalon-Dangur I., Nashashibi J., Yahav D., Paul M., Leibovici L. (2022). Underrepresentation
of Women in Randomized Controlled Trials: A Systematic Review and
Meta-Analysis. Trials.

[49] Sedano R., Hogan M., Mcdonald C., Aswani-Omprakash T., Ma C., Jairath V. (2022). Underrepresentation of Minorities and Underreporting
of Race and Ethnicity in Crohn’s Disease Clinical Trials. Gastroenterology.

[50] King Z. A., Lu J., Dräger A., Miller P., Federowicz S., Lerman J. A., Ebrahim A., Palsson B. O., Lewis N. E. (2016). BiGG Models:
A Platform for Integrating, Standardizing and Sharing Genome-Scale
Models. Nucleic Acids Res..

[51] Bzdok D., Altman N., Krzywinski M. (2018). Statistics
versus Machine Learning. Nat. Methods.

[52] Shao Z., Wang F., Xu Y., Wei W., Yu C., Zhang Z., Yao D., Sun T., Jin G., Cao X., Cong G., Jensen C. S., Cheng X. (2025). Exploring
Progress
in Multivariate Time Series Forecasting: Comprehensive Benchmarking
and Heterogeneity Analysis. IEEE Transactions
on Knowledge and Data Engineering.

[53] Murdoch W. J., Singh C., Kumbier K., Abbasi-Asl R., Yu B. (2019). Definitions, Methods, and Applications
in Interpretable Machine Learning. Proceedings
of the National Academy of Sciences USA.

[54] Sidak D., Schwarzerová J., Weckwerth W., Waldherr S. (2022). Interpretable Machine
Learning Methods for Predictions in Systems Biology from Omics Data. Frontiers in Molecular Biosciences.

