# Supplementary material for: Simulation of the Metabolic Response to an Interventional Study with New Healthy Beverages by Machine-Learning Regression
Source: J Agric Food Chem. 2026 Mar 14;74(11):9683–92. doi: 10.1021/acs.jafc.5c15421 (PMC13022870; doi:10.1021/acs.jafc.5c15421)
Supplement: Supplementary file 1 [file jf5c15421_si_001.pdf]

Supporting information for

Simulation of the Metabolic Response to an Interventional Study with New Healthy Beverages by Machine Learning Regression

Diego Hernández-Prieto [1], Jose A. Egea [2,*], Cristina García-Viguera [1,3], Alberto Garre [3,4]

1. Lab Fitoquimica y Alimentos Saludables (LabFAS), CEBAS-CSIC, Campus Universitario Espinardo 25, 30100 Murcia, Spain; dprieto@cebas.csic.es, cgviguera@cebas.csic.es

2. Group of Fruit Breeding, Department of Plant Breeding, CEBAS-CSIC, Campus Universitario de Espinardo 25, 30100 Murcia, Spain; jaegea@cebas.csic.es

3. Associated Unit of R&D and Innovation CEBAS-CSIC+UPCT on "Quality and Risk Assessment of Foods", Campus Universitario Espinardo 25, 30100 Murcia, Spain;

4. Departamento de Ingeniería de Alimentos y del Equipamiento Agrícola, Instituto de Biotecnología Vegetal, Universidad Politécnica de Cartagena (ETSIA), Paseo Alfonso XIII, 48, 30203 Cartagena, Spain; alberto.garre@upct.es

*corresponding author: jaegea@cebas.csic.es

Supplementary Table 1. Metabolites found and quantified in the interventional trial.

Family of compounds	Sample	Metabolites
Anthocyanins metabolites	Plasma & Urine	CA, CA-G, CA-GS, Total CA, DHPAA, DHPAA-G, DHPAA-GG, DHPAA-GS, DHPAA-SS, Total DHPAA, TFA-G, TFA-S, Total TFA, VA, VA-GG, VA-SS, VA-GS, Total VA
	Only Urine	VA-S
Flavanones and their metabolites	Plasma & Urine	E, E-S, Total E, N-G
	Only Plasma	HE-G
	Only Urine	E-G, HE, HE-GG, Total HE, N, N-GG, N-S, Total N

Supplementary Table 2. List of hyperparameters considered in Bayesian optimization procedure to be tuned for each model with its range assessed.

Model	Hyperparameter	Description & Range of values
RF	*n_estimators*	The number of trees in the forest (10,1000)
	min_samples_leaf	The minimum number of samples required to be at a leaf node (1,50)
	colsample_bytree	The fraction of features to be considered when looking for the best split in each tree (0.3, 1)
	colsample_bynode	The fraction of features to be considered when looking for the best split at each node (0.1, 1)
	subsample	The fraction of samples to be used for fitting each individual tree, introducing randomness, and reducing overfitting (0.1, 1)
XGBoost	*n_estimators*	The number of boosting rounds, or trees, to be added to the model (10,1000)
	max_depth	The maximum depth of each decision tree, controlling the complexity and risk of overfitting (3,12)
	learning_rate	The step size shrinkage to prevent overfitting by reducing the impact of each tree. (0.00001, 0.01)
	reg_lambda	L2 regularization term on weights, which can help reduce overfitting. (0.0001, 0.1)
	reg_alpha	L1 regularization term on weights, which can help with feature selection and reduce overfitting (0.0001, 0.1)
	colsample_bynode	The subsample ratio of columns for each split, controlling the number of features considered (0.1, 1)
	subsample	The subsample ratio of the training data before growing trees, reducing overfitting by introducing randomness (0.1, 1)
LightGBM	*n_estimators*	The number of boosting rounds or trees in the model (100,1000)
	max_depth	The maximum depth of each tree to prevent overfitting (-1,10)
	learning_rate	The step size shrinkage to prevent overfitting by reducing the impact of each trees (0.0001, 0.1)
	subsample	The fraction of data to be used for each iteration, introducing randomness, and reducing overfitting (0.01, 1)

	boosting_type	The type of boosting to use ('gbdt' for traditional Gradient Boosting Decision Tree, 'dart' for Dropouts meet Multiple Additive Regression Trees)
	num_leaves:	The maximum number of leaves in each tree, controlling the complexity and depth of the tree (1024, 2000).

Supplementary Table 3. Metrics obtained for the best performance model for every metabolite considered. The goodness of fit of models was reflected in R^2 and the accuracy of the predictions was expressed in Mean Absolute Error (MAE).

Target metabolite	Selected algorithm	R^2	MAE (ng/ml)
VA (urine)	XGB	0,935	4,36 x 10^{-3}
VA (plasma)	XGB	0,948	2,03 x 10^{-2}
VA-S	XGB	0,742	5,09 x 10^{-2}
VA-SS (urine)	XGB	0,921	5,68 x 10^{-3}
VA-SS (plasma)	XGB	0,905	2,66 x 10^{-2}
VA-GG (urine)	XGB	0,828	2,45 x 10^{-2}
VA-GG (plasma)	XGB	0,930	1,09 x 10^{-2}
VA-GS (urine)	XGB	0,921	8,85 x 10^{-3}
VA-GS (plasma)	XGB	0,915	3,64 x 10^{-2}
Total VA (urine)	XGB	0,997	1,23 x 10^{-3}
Total VA (plasma)	XGB	0,958	1,55 x 10^{-2}
TFA-S (urine)	XGB	0,991	5,33 x 10^{-3}
TFA-S (plasma)	RF	0,834	8,76 x 10^{-3}
TFA-G (urine)	XGB	0,844	3,44 x 10^{-3}
TFA-G (plasma)	XGB	0,730	1,43 x 10^{-2}
Total.TFA (urine)	XGB	0,996	4,01 x 10^{-3}
Total.TFA (plasma)	XGB	0,960	4,47 x 10^{-3}
HE	LGBM	0,961	1,13 x 10^{-2}
HE-G (urine)	XGB	0,842	1,05 x 10^{-2}
HE-G (plasma)	XGB	0,954	2,03 x 10^{-3}
HE-GG	XGB	0,991	6,18 x 10^{-3}
Total HE	RF	0,894	9,59 x 10^{-3}
N	RF	0,952	4,09 x 10^{-3}
N-G (urine)	XGB	0,982	5,38 x 10^{-3}
N-G (plasma)	XGB	0,977	5,06 x 10^{-3}
N-S	XGB	0,972	7,31 x 10^{-3}
N-GG	RF	0,860	7,80 x 10^{-3}
Total N	XGB	0,984	4,93 x 10^{-3}
E (urine)	RF	0,917	1,01 x 10^{-2}

E (plasma)	XGB	0,867	3,43 x 10^{-2}
E-G	XGB	0,872	7,36 x 10^{-3}
E-S (urine)	LGBM	0,842	3,86 x 10^{-2}
E-S (plasma)	XGB	0,987	1,76 x 10^{-2}
Total E (urine)	XGB	0,905	5,55 x 10^{-3}
DHPAA (urine)	XGB	0,916	1,95 x 10^{-2}
DHPAA (plasma)	XGB	0,868	4,37 x 10^{-2}
DHPAA SS (urine)	RF	0,946	5,59 x 10^{-3}
DHPAA G (urine)	XGB	0,976	7,17 x 10^{-3}
DHPAA G (plasma)	XGB	0,989	9,41 x 10^{-3}
DHPAA GS (urine)	XGB	0,941	1,76 x 10^{-2}
DHPAA GS (plasma)	XGB	0,886	2,53 x 10^{-2}
DHPAA GG (urine)	RF	0,781	1,20 x 10^{-2}
DHPAA GG (plasma)	XGB	0,968	1,33 x 10^{-2}
Total DHPAA (urine)	XGB	0,995	3,42 x 10^{-3}
Total DHPAA (plasma)	RF	0,876	3,63 x 10^{-2}
CA (urine)	XGB	0,887	3,20 x 10^{-3}
CA (plasma)	XGB	0,981	1,34 x 10^{-2}

Supplementary Table 4. Evaluation of effects of biochemical family and sample type factors (along with their interactions) on the accuracy obtained by studied models, measured using a mixed-ANOVA. Bold values represent a *p*-value < 0.05

Metric	Family	Sample	Family:Sample
R^2	3.51 x 10^{-1}	4.314 x 10^{-1}	2.45 x 10^{-1}
MAE (ng/ml)	4.65 x 10^{-1}	**2 x 10^{-4}**	**4.96 x 10^{-2}**

Supplementary Table 5. Descriptive statistical values for each concentration of bioactive compound. Units for all reported concentrations (i.e, mean, median, maximum, minimum and Q1 and Q3 quantiles) are expressed in ng/ml. pre_i: pre-intervention; post_i: post-intervention

Compound	Time	Sample	mean	median	max	min	Q1	Q3
VA	pre_i	urine	24,396	12,199	198,366	0,528	5,978	27,002
VA	post_i	urine	41,118	17,708	600,304	0,190	8,882	39,840
VA	pre_i	plasma	3,118	3,139	6,259	1,369	2,526	3,427
VA	post_i	plasma	4,380	3,810	13,162	1,369	2,829	5,527
VA-SS	pre_i	urine	1,762	1,240	26,793	0,001	0,622	2,162
VA-SS	post_i	urine	1,349	0,968	5,820	0,003	0,455	1,981

VA-SS	pre_i	plasma	6,655	5,597	25,655	1,825	3,940	8,392
VA-SS	post_i	plasma	8,916	6,834	29,033	1,931	5,012	12,255
VA-GS	pre_i	plasma	9,414	8,227	26,282	2,267	6,225	12,399
VA-GS	post_i	plasma	12,105	11,247	28,120	2,785	7,226	16,144
Total_VA	pre_i	urine	31,193	20,484	203,866	0,000	12,016	34,753
Total_VA	post_i	urine	47,448	24,847	603,660	4,136	14,345	49,226
Total VA	pre_i	plasma	45,218	41,821	193,770	13,713	32,818	53,406
Total VA	post_i	plasma	78,580	68,006	244,209	33,881	54,503	90,142
TFA-Sulfate	pre_i	urine	129,156	74,815	866,516	0,524	38,770	164,873
TFA-Sulfate	post_i	urine	131,595	99,364	924,052	2,490	47,053	160,271
TFA-Gluc	pre_i	urine	0,724	0,314	29,269	0,052	0,189	0,575
TFA-Gluc	post_i	urine	0,617	0,319	4,093	0,059	0,213	0,728
Total_TFA	pre_i	urine	125,327	71,451	867,718	0,000	36,600	159,830
Total_TFA	post_i	urine	131,078	99,044	924,289	1,046	47,545	159,575
Total_TFA	pre_i	plasma	7,232	3,647	157,779	0,000	1,659	6,704
Total_TFA	post_i	plasma	11,899	6,740	205,538	0,000	3,224	12,018
HE	pre_i	urine	0,108	0,092	0,414	0,036	0,071	0,118
HE	post_i	urine	0,103	0,092	0,282	0,015	0,066	0,122
HE-G	pre_i	urine	1,953	0,245	91,600	0,000	0,097	0,621
HE-G	post_i	urine	5,480	1,546	40,742	0,018	0,531	5,751
HE-G	pre_i	plasma	8,719	2,626	35,300	0,100	0,975	13,650
HE-G	post_i	plasma	34,493	6,000	683,200	0,300	2,700	17,700
HE-GG	pre_i	urine	0,111	0,099	0,360	0,035	0,070	0,122
HE-GG	post_i	urine	0,107	0,091	0,319	0,017	0,067	0,129
Total HE	pre_i	urine	1,892	0,397	92,308	0,000	0,239	0,717
Total HE	post_i	urine	5,103	1,472	41,151	0,000	0,501	4,848
N	pre_i	urine	2,050	0,996	12,694	0,440	0,308	2,330
N	post_i	urine	2,375	1,072	58,106	0,001	0,390	2,161
N-G	pre_i	urine	86,130	44,961	1503,575	2,552	23,883	87,097
N-G	post_i	urine	125,870	83,682	616,175	3,742	42,338	179,337
N-G	pre_i	plasma	138,384	118,174	719,065	4,331	72,070	174,619
N-G	post_i	plasma	246,661	182,598	3215,856	42,313	123,493	253,331
N-S	pre_i	urine	12,355	8,469	81,979	0,890	4,702	14,882
N-S	post_i	urine	13,278	7,684	124,403	1,509	4,415	14,533
Total N	pre_i		101,809	59,770	1577,335	8,073	37,569	100,840
Total N	post_i		141,267	97,341	624,055	6,873	48,288	192,965
E	pre_i	urine	0,009	0,006	0,097	0,002	0,004	0,010
E	post_i	urine	0,010	0,008	0,073	0,001	0,005	0,013
E	pre_i	plasma	0,357	0,333	0,988	0,130	0,249	0,427
E	post_i	plasma	0,428	0,406	0,942	0,120	0,318	0,525
E-G	pre_i	urine	9,362	4,514	82,264	0,112	1,525	11,862
E-G	post_i	urine	9,976	4,227	266,512	0,058	1,528	10,689
CA	pre_i	urine	0,025	0,014	0,566	0,002	0,008	0,023

CA	post_i	urine	0,026	0,019	0,185	0,001	0,011	0,030
CA	pre_i	plasma	0,310	0,288	0,796	0,128	0,223	0,375
CA	post_i	plasma	0,432	0,410	0,963	0,191	0,318	0,535
VA-GG	pre_i	urine	1,539	1,233	8,105	0,020	0,514	2,057
VA-GG	post_i	urine	1,346	1,103	6,108	0,058	0,538	1,973
VA-GS	pre_i	urine	5,682	4,449	29,853	0,537	2,630	6,586
VA-GS	post_i	urine	4,568	3,623	16,785	0,212	2,309	5,788
DHPAA	pre_i	urine	0,265	0,216	1,347	0,005	0,124	0,345
DHPAA	post_i	urine	0,367	0,288	2,133	0,020	0,123	0,476
DHPAA	pre_i	plasma	10,155	9,649	33,917	1,923	6,659	12,466
DHPAA	post_i	plasma	14,717	13,459	33,321	3,953	10,300	18,395
DHPAA-SS	pre_i	urine	0,149	0,108	2,512	0,002	0,045	0,173
DHPAA-SS	post_i	urine	0,116	0,083	0,512	0,008	0,046	0,166
DHPAA-G	pre_i	urine	2,419	1,567	15,067	0,094	0,799	2,895
DHPAA-G	post_i	urine	3,162	1,745	38,481	0,021	0,916	3,551
DHPAA-G	pre_i	plasma	1,244	1,105	9,439	0,084	0,528	1,567
DHPAA-G	post_i	plasma	2,050	1,564	8,635	0,235	0,864	2,624
DHPAA-GS	pre_i	urine	0,130	0,101	0,762	0,001	0,039	0,186
DHPAA-GS	post_i	urine	0,119	0,089	0,536	0,001	0,049	0,168
DHPAA-GS	pre_i	plasma	0,577	0,475	2,642	0,001	0,266	0,728
DHPAA-GS	post_i	plasma	0,823	0,679	3,921	0,114	0,420	1,025
DHPAA-GG	pre_i	plasma	1,536	1,006	7,318	0,041	0,421	1,998
DHPAA-GG	post_i	plasma	2,861	1,867	15,560	0,075	0,951	3,627
Total DHPAA	pre_i	urine	2,785	1,883	16,648	0,000	1,216	3,231
Total DHPAA	post_i	urine	3,709	2,489	38,836	0,230	1,320	4,480
Total DHPAA	pre_i	plasma	13,883	13,379	45,905	4,931	10,054	16,559
Total DHPAA	post_i	plasma	21,030	19,085	47,694	7,131	15,411	25,312
VA-S	pre_i	plasma	6,220	5,571	19,777	1,369	4,505	7,445
VA-S	post_i	plasma	8,857	7,310	30,112	3,403	5,777	10,400
VA-GG	pre_i	plasma	25,466	20,841	165,989	2,081	13,965	31,814
VA-GG	post_i	plasma	46,868	38,189	205,800	9,477	26,839	56,260
E-S	pre_i	plasma	0,218	0,196	0,674	0,088	0,138	0,258
E-S	post_i	plasma	0,229	0,196	1,056	0,088	0,154	0,263
E-S	pre_i	urine	0,088	0,064	0,547	0,008	0,035	0,101
E-S	post_i	urine	0,091	0,063	0,544	0,012	0,045	0,118
Total E	pre_i	urine	0,437	0,421	1,035	0,000	0,285	0,580
Total E	post_i	urine	0,541	0,556	1,230	0,000	0,373	0,714
TFA-S	pre_i	plasma	6,099	2,399	151,197	0,044	1,159	5,341
TFA-S	post_i	plasma	9,822	4,657	169,101	0,018	2,508	9,446
TFA-G	pre_i	plasma	2,079	1,270	11,098	0,015	0,550	2,789
TFA-G	post_i	plasma	3,078	1,719	36,437	0,041	0,560	3,333

N-GG	pre_i	urine	1,858	0,833	45,475	0,040	0,296	1,898
N-GG	post_i	urine	1,242	0,578	10,020	0,001	0,283	1,285

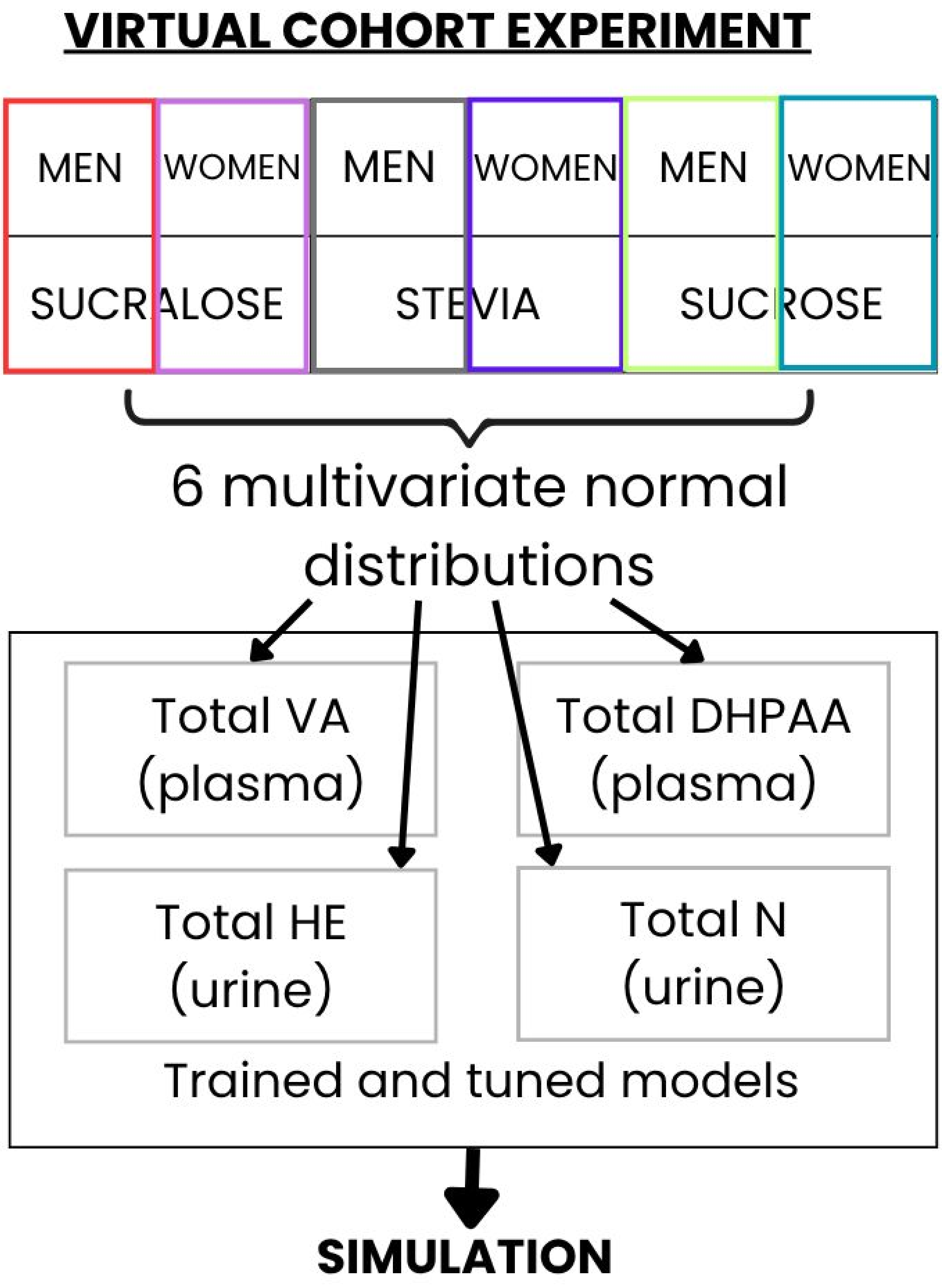

Supplementary Figure 1. Scheme of the virtual cohort for the simulation experiments.

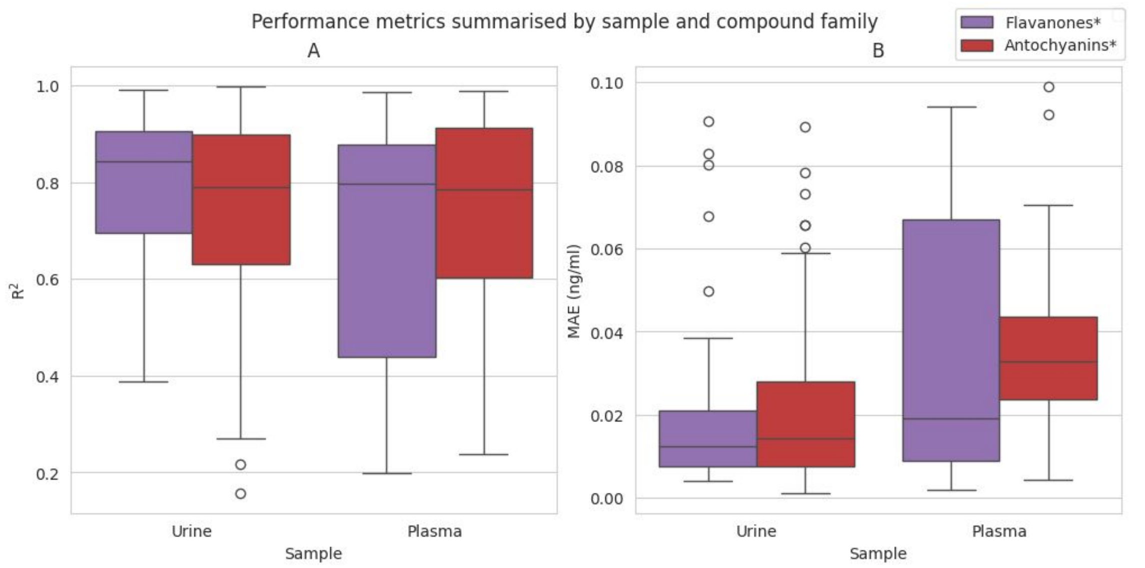

Supplementary Figure 2. Boxplot of values obtained in performance metrics by the two families considered.
